# Factores de riesgo de deterioro cognitivo asociado a cáncer en pacientes con carcinoma de mama y colon que reciben tratamiento con quimioterapia

**DOI:** 10.23938/ASSN.1040

**Published:** 2023-08-16

**Authors:** Patricia Iranzo, Ana Callejo, Julio Arbej, Sebastian Menao, Dolores Isla, Raquel Andrés

**Affiliations:** 1 Hospital Universitari Vall d'Hebron Servicio de Oncología Médica Hospital Universitario Vall d’Hebron Barcelona Spain; 2 Vall d’Hebron Institute of Oncology (VHIO) Barcelona España; 3 Hospital Clínico Universitario Lozano Blesa Servicio de Psiquiatría Hospital Clínico Universitario Lozano Blesa Zaragoza Spain; 4 Hospital Clínico Universitario Lozano Blesa Servicio de Bioquímica Hospital Clínico Universitario Lozano Blesa Zaragoza Spain; 5 Hospital Clínico Universitario Lozano Blesa Servicio Oncología Médica Hospital Clínico Universitario Lozano Blesa Zaragoza Spain; 6 Instituto de Investigación Sanitaria de Aragón Zaragoza España

**Keywords:** Disfunción cognitiva, Neoplasia, Factor de riesgo, Quimioterapia, Polimorfismo genético, Cognitive impairment, Cancer, Chemotherapy, Risk factor, Single nucleotide polymorphism

## Abstract

**Fundamento:**

Nuestro estudio se plantea con el objetivo de evaluar el impacto de diferentes factores individuales sobre el deterioro cognitivo relacionado con el cáncer en pacientes tratados con quimioterapia.

**Material y métodos:**

Estudio unicéntrico longitudinal prospectivo. Incluyó pacientes con carcinoma de mama y colon tratados con quimioterapia. Se recogieron variables clínicas y genéticas del paciente (polimorfismos de nucleótido simple, SNP). Los pacientes fueron evaluados neurocognitivamente con once test validados, en tres momentos: basal previo a quimioterapia (M0), entre una y cuatro semanas tras finalizar quimioterapia (M1) y entre 24-30 semanas tras finalizar quimioterapia (M2).

**Resultados:**

Se incluyeron 62 pacientes, 82% mujeres, con mediana de edad de 56 años (rango 30-74), un 64,5% con cáncer de mama. La edad <55 años, tener estudios superiores, ausencia de comorbilidades y presencia de la variante CC de rs471692 (TOP2A) se asociaron, en general, con mejores resultados cognitivos en M0. Se observó un empeoramiento significativo de M0 a M1 en los test RAVLT y Letras y números, y recuperación en M2 respecto a M0 en los test de memoria visual, FAST, clave de números, y cubos. La edad ≥55 años, la quimioterapia adyuvante, las comorbilidades, el consumo de tabaco y de alcohol y la variante GT de rs1800795 se relacionaron con el deterioro entre M0 y M1 en el modelo multivariante.

**Conclusiones:**

La edad mayor de 55 años, el sexo femenino, la presencia de comorbilidades y el nivel básico de estudios se relacionan con un mayor riesgo de deterioro cognitivo tras el tratamiento con quimioterapia.

## Abstract (en)

**Background:**

Our study aims to evaluate the impact of different factors on cancer-related cognitive impairment in patients who undergo chemotherapy.

**Methodology:**

Prospective longitudinal single-centre study that included patients with breast and colon carcinoma who underwent chemotherapy as part of their treatment. Clinical and genetic characteristics of the patients (single nucleotide polymorphisms, SNPs) were collected. Patients’ neurocognitive status was assessed using eleven validated tests at three time points: before chemotherapy (M0 - baseline), between one and four weeks after completing chemotherapy (M1), and between 24-30 weeks after completing chemotherapy (M2).

**Results:**

Sixty-two patients were included in this study; 82% were female, median age was 56 years (range 30-74), and 64.5% had been diagnosed with breast cancer. Overall, better cognitive results at M0 were associated with age < 55 years, higher educational level, absence of comorbidities, and the CC variant rs471692 (TOP2A). Significant decline was found between M0 to M1 in the Rey Auditory Verbal Learning Test and the Letter and Number test, with evidence of recovery in M2 compared to M0 regarding the following test: Visual Memory, Functioning Assessment Short Test (FAST), Digit Symbol Substitution and Cube. In the multivariate analysis, being ≥55 years of age, adjuvant chemotherapy, presence of comorbidities, tobacco and alcohol use, and GT variant rs1800795 were associated with cognitive decline between M0 and M1.

**Conclusion:**

Being ≥55 years of age, female, presence of comorbidities and basic education level are related to a higher risk of cognitive impairment after chemotherapy.

## INTRODUCCIÓN

La supervivencia global de los pacientes con tumores sólidos está aumentando de manera progresiva en los últimos años y, de manera paralela, se describen alteraciones neurocognitivas en estos pacientes cada vez con mayor frecuencia^1^. Un 30% de las personas diagnosticadas de tumores sólidos presentan problemas cognitivos incluso antes de iniciar cualquier terapia antineoplásica, y hasta el 75% de las que reciben quimioterapia registran empeoramiento de sus funciones cognitivas^2^.

Por ello, se ha acuñado el término *deterioro cognitivo relacionado con el cáncer* (CRCI) para referirse a esta entidad que afecta a diversos dominios^3^. La intensidad, duración y los dominios afectados son diferentes entre individuos^4^, y el espectro de alteraciones y su severidad varía de manera importante. Además, no todas las personas con diagnóstico de neoplasia sometidas a tratamiento oncológico desarrollan CRCI.

La evaluación de la capacidad cognitiva junto con la identificación de aquellos factores que hacen a un paciente más vulnerable a las alteraciones del sistema nervioso, condicionadas por la enfermedad y su tratamiento, puede contribuir a identificar los cambios cognitivos en una fase temprana, a valorar el impacto sobre la calidad de vida y, como consecuencia, orientar a los facultativos en las estrategias de prevención, seguimiento y potencial tratamiento.

Para una adecuada evaluación del CRCI, *The International Cognition and Cancer Task Force* (ICCTF)^5^ recomienda incluir en el plan de evaluación neurocognitiva una evaluación basal previa al inicio del tratamiento y, posteriormente, realizar evaluaciones seriadas para poder identificar los cambios a lo largo del tiempo en los siguientes dominios: Memoria y aprendizaje, Función ejecutiva, y Velocidad de procesamiento y coordinación. La evaluación basal es especialmente importante para detectar aquellos pacientes que, tras el diagnóstico y/o el tratamiento, mantienen sus funciones cognitivas dentro de los niveles estándar pero mostrando un deterioro respecto a su situación basal^6^.

Entre los posibles factores que pueden predisponer al desarrollo de CRCI se han descrito factores sociodemográficos, psicológicos, y médicos; especialmente la edad avanzada, la reserva cognitiva baja, el nivel educativo básico, y la presencia de ansiedad, depresión y comorbilidades (como diabetes mellitus, patología cardiovascular e insuficiencia renal)^7^^-^^9^.

Las diferencias individuales en polimorfismos de nucleótido simple (*single nucleotide polymorphism*, SNP) también han sido incluidas como potenciales factores de riesgo en el desarrollo de CRCI^10^. La mayoría de los estudios que incluyen análisis de SNP se centran en aquellos directamente relacionados con los fenómenos de reparación y plasticidad neuronal (como BDNF, ApoE y COMT), aunque también se han identificado genes implicados en la neurotransmisión y la respuesta inflamatoria como potenciales factores asociados al deterioro cognitivo^11^. Las frecuencias relativamente bajas de determinados polimorfismos y su desigual distribución geográfica suponen un reto para realizar estudios que permitan extraer conclusiones robustas.

Bajo la hipótesis de que diferentes factores clínicos y genéticos pueden aumentar el riesgo de CRCI, se realiza el presente trabajo con el objetivo de identificar, en la práctica clínica, a pacientes con cáncer con mayor predisposición a CRCI. Para ello, evaluamos el impacto de distintas variables sobre los resultados de una batería de test neurocognitivos antes y después de recibir quimioterapia.

## MATERIAL Y MÉTODOS

Se realizó un estudio prospectivo longitudinal en el Servicio de Oncología Médica del Hospital Clínico Universitario Lozano Blesa (Zaragoza, España), entre 1 de junio de 2017 y el 31 diciembre de 2019. El proyecto fue aprobado por el Comité de Ética de la Investigación de la Comunidad de Aragón (actas CP04/2016 y 10/2017).

Se incluyeron consecutivamente pacientes de entre 18 y 75 años diagnosticados de carcinoma infiltrante de mama o carcinoma de colon candidatos a recibir tratamiento con quimioterapia adyuvante o neoadyuvante.

Se excluyeron aquellos pacientes con exposición en los cinco años previos a quimioterapia o radioterapia, con historia previa de enfermedad neurológica, abuso de sustancias, traumatismo craneal o diagnóstico de enfermedad psiquiátrica de acuerdo al *Diagnostic and Statistical Manual of Mental Disorders-5.*

De cada paciente se recogieron las siguientes variables:

- *sociodemográficas*: edad (años cumplidos; <55, ≥55), sexo (hombre, mujer), nivel de estudios (primaria/secundaria, superiores: bachillerato, formación profesional o universitarios), estado civil (casado, otros), apoyo social percibido (sí, no);
- *clínicas*: presencia de comorbilidades (sí, no), número de comorbilidades (<2, ≥2), diabetes (DM) (sí, no), hipertensión (HTA) (sí, no), situación funcional (asintomática, con síntomas), tratamiento concomitante (sí, no), número de fármacos (<2, ≥2), tabaquismo (sí, no), consumo de alcohol (sí, no);
- *relacionadas con la neoplasia*: tipo de tumor (mama, colon);
- *relacionadas con el tratamiento*: tipo de quimioterapia (neoadyuvante, adyuvante), radioterapia adyuvante (en cáncer de mama) (sí, no), hormonoterapia (en cáncer de mama) (sí, no).

### Evaluaciones neurocognitivas

Personal cualificado y entrenado realizó las evaluaciones neurocognitivas en tres momentos diferentes:

- Basal (M0): entre 4 semanas y 48 horas antes al inicio del tratamiento con quimioterapia.
- Post-quimioterapia (M1): entre una y cuatro semanas tras la finalización del tratamiento con quimioterapia.
- De seguimiento a largo plazo (M2): entre 24 y 30 semanas tras la finalización del tratamiento con quimioterapia.

Se emplearon once pruebas de evaluación neurocognitiva: test de Aprendizaje Auditivo-Verbal de Rey (RAVLT)^12^, Clave de números, Letras-Números y Cubos de Wechsler para adultos-IV^13^, Test de Dígitos de Wechsler para adultos-III^14^, Fluencia Verbal Semántica Animales de la batería de evaluación de la Memoria Semántica, Subtest de Fluencia Verbal Fonética (FAS) y de memoria visual inmediata de Test de Barcelona^15^, Test de STROOP^16^ y *Trail Making Test* (TMT) A y B^17^. Con estos test se exploran las siguientes funciones:

- Memoria verbal y visual: RAVLT (número total de palabras recordadas: de 0 a 45, relación directa), letras-números (puntuación estimada de 1 a 19, directa), test de dígitos (puntuación estimada de 1 a 19, directa), y test de memoria visual (percentil ajustado por edad, directa).
- Función ejecutiva: test de Stroop (capacidad de resistir interferencia verbal, directa), test de fluencia verbal fonética y semántica *(functioning assessment short test,* FAST) (número total de palabras, directa), letras-números y TMT-B (tiempo empleado en segundos, inversa).
- Atención y función psicomotora: test de dígitos, clave de números (puntuación estimada de 1 a 19, directa) y TMT-A.
- Función visoconstructiva: test de cubos (puntuación estimada de 1 a 19, directa).

Por tanto, en todos los test, mayor puntuación implica mejor funcionalidad excepto en TMT-A y TMT-B, en los que mayor puntuación implica peor capacidad. Las puntuaciones estimadas se obtienen según la conversión expuesta en el manual de cada prueba de acuerdo a la edad y el nivel de estudios de los participantes.

### Análisis de polimorfismos de nucleótido simple (SNP)

En M2 se seleccionaron las personas participantes en el subestudio de SNP entre aquellas que habían completado las tres evaluaciones. Previa firma de un consentimiento informado específico, se obtuvo la muestra de sangre periférica total de la que se consiguió el ADN que se utilizó como molde para amplificar y estudiar los SNP.

Los SNP estudiados se seleccionaron según la evidencia científica actual:

- *Ankyrin repeat and Kinase domain containing 1* (*ANKK1)* (rs1800497): el alelo T de este polimorfismo se ha asociado con deterioro cognitivo por lo que se agrupa a los pacientes en función de la presencia o ausencia de este alelo T^18^.
- Factor de necrosis tumoral (TNF-α-308 G>A) (rs1800629): el genotipo *AG* se ha asociado con peores resultados en test de memoria en pacientes con cáncer de mama^19^.
- Interleuquina-6 (IL-6) (rs1800795 T>G): los individuos con variante *GG* presentan más riesgo de alteraciones de la atención^20^.
- Topoisomerasa 2A (TOP2A) (rs471692 T>C): el genotipo *CC* se ha relacionado con deterioro de atención tras tratamiento quimioterápico^21^.
- Factor neurotrófico derivado del cerebro (BDNF) (rs6265 G>A): la variante *GA* se ha asociado a posible efecto protector frente a CRCI^22^.
- Apolipoproteína E (APOE) (rs429358): el alelo Ɛ4 de APOE se ha identificado como factor de riesgo de CRCI^23^.

Todos los genotipados se llevaron a cabo en el Centro Nacional de Genotipado, Plataforma de Recursos Biomoleculares del Instituto de Salud Carlos III (CeGen-PRB3-ISCIII), a excepción del genotipado de rs429358, realizado en el Laboratorio de Bioquímica de nuestro centro mediante secuenciación Sanger.

### Análisis estadístico

Las variables categóricas se describieron como frecuencias y porcentajes, y las cuantitativas como mediana ± rango intercuartílico, tras realización de la prueba de normalidad (Kolmogorov-Smirnov o Shapiro-Wilk). Se utilizó la prueba U de Mann-Whitney para estudiar la asociación entre los resultados neurocognitivos y las variables identificadas como posibles factores de riesgo (edad, sexo, nivel estudios, comorbilidades, tabaco, alcohol, tipo de quimioterapia, radioterapia y SNP).

Se realizó un análisis de regresión lineal múltiple por pasos hacia atrás, utilizando la puntuación en la prueba como variable dependiente e incluyendo las variables del estudio como variables independientes.

Para estudiar los cambios neurocognitivos potencialmente mediados por la administración de quimioterapia se calculó la diferencia entre las puntuaciones obtenidas por cada sujeto en los diferentes momentos de cada una de las pruebas y se analizó mediante pruebas para muestras relacionadas: t de Student para variables de distribución normal y test de Wilcoxon para aquellas sin normalidad.

Con el objetivo de evaluar el papel de la quimioterapia en la función cognitiva, se analizó de forma multivariante la diferencia de las puntuaciones entre M0 y M1 (puntuación obtenida en M0 menos puntuación obtenida en M1) para aquellas variables con mayor impacto en las puntuaciones de los test en M0 y M1.

El análisis estadístico se realizó con el programa SPSS versión 25.0, considerándose un resultado estadísticamente significativo (rechazo de la hipótesis nula) si *p* < 0,05.

## RESULTADOS

**Figura 1:**
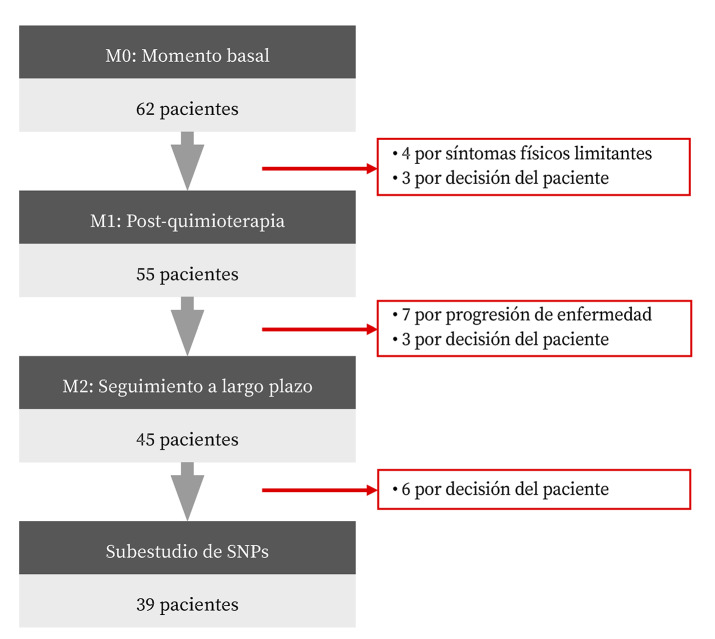
Sujetos incluidos en el estudio y pérdidas durante el seguimiento.

Se incluyeron un total de 62 pacientes. La figura 1 muestra el número de sujetos incluidos en cada una de las evaluaciones con las pérdidas producidas y el motivo de las mismas. La mediana de edad fue 56 años (rango 30 a 74). El 82,3% eran mujeres; la mediana de edad en el grupo de mujeres fue de 55 años (rango 30 a 74), y en hombres 62 años (rango 51 a 72).

El 58,1% de pacientes presentaban comorbilidades en el momento de la inclusión en el estudio, la mayoría (67,7%) menor de 2, y más del 80% eran funcionalmente asintomáticos. La mitad tomaban tratamiento para sus comorbilidades, dos o más fármacos en casi el 40% de los casos (Tabla 1).

La mayoría de las pacientes (64,5%) tenían diagnóstico de carcinoma de mama y recibieron quimioterapia adyuvante.

El subestudio de SNP incluyó 39 participantes (62,9%) con una mediana de edad de 55 años (rango 30 a 72); el 82% eran mujeres y más de la mitad (59%) tenían estudios superiores. Este subgrupo mostró la misma distribución de variables que la cohorte global, excepto que las pacientes con cáncer de mama recibieron menos radioterapia (51,3 vs 77,5%) y menos hormonoterapia (39 vs 59%) (Tabla 1).

**Tabla 1:** Variables sociodemográficas y clínicas en la cohorte global y en subestudio de SNP (n=39)

	Global	Subgrupo SNP
	n = 62	n = 39
	n (%)	n (%)
*Variable*		
*Edad*		
Mediana (RIC)	56 (15)	55.5 (11)
Rango	30-74	30-74
≥55 años	26 (41,9)	18 (46,2)
*Sexo*		
Hombre	11 (17,7)	7 (18)
Mujer	51 (82,3)	32 (82)
*Nivel estudios*		
Primaria/secundaria	31 (50)	16 (41)
Superiores	31 (50)	23 (59)
*Estado civil*		
Casado	49 (79)	22 (56,4)
Otros	13 (21)	17 (43,6)
*Comorbilidades*		
Sí	36 (58,1)	21 (53,8)
No	26 (41,9)	18 (46,2)
*Número comorbilidades*		
< 2	42 (67,7)	27 (69,2)
≥ 2	20 (32,3)	12 (30,8)
*Diabetes*		
Sí	9 (14,5)	6 (15,4)
No	53 (85,5)	33 (84,6)
*Hipertensión*		
Sí	15 (24,2)	7 (18)
No	47 (75,8)	32 (82)
*Situación funcional*		
Asintomático	51 (82,3)	31 (79,5)
Con síntomas	11 (17,7)	8 (20,5)
*Tratamiento concomitante*		
Sí	31 (50)	17 (43,6)
No	31 (50)	22 (56,4)
*Número de fármacos*		
< 2	38 (61,3)	26 (66,7)
≥ 2	24 (38,7)	13 (33,3)
*Consumo de tabaco*		
Sí	13 (21)	10 (25,6)
No	49 (79)	29 (74,4)
*Consumo de alcohol*		
Sí	12 (19,4)	9 (26,1)
No	50 (80,7)	30 (76,9)
*Apoyo social percibido*		
Sí	57 (91,9)	37 (94,9)
No	5 (9,1)	2 (5,1)
*Tipo de tumor*		
Mama	40 (64,5)	26 (66,7)
Colon	22 (35,5)	13 (33,3)
*Quimioterapia*		
Neoadyuvante	20 (32,3)	13 (33,3)
Adyuvante	42 (67,7)	26 (66,7)
*Radioterapia adyuvante (mama n=40)*		
Sí	31 (77,5)	20 (51,3)
No	9 (22,5)	19 (48,7)
*Hormonoterapia (mama n=40)*		
Sí	26 (65)	15 (39)
No	14 (35)	24 (61)
SNP		
*rs1800497 (ANKK1)*		
T presente		15 (38,5)
T ausente		24 (61,5)
*rs1800629 (TNFα)*		
GG		25(64,1)
AG		14 (35,9)
*rs1800795 (IL-6)*		
GT		17 (43,6)
GG		22 (56,4)
*rs471692 (TOP2A)*		
CT		9 (23,1)
CC		30 (76,9)
*rs6265 (BDNF)*		
GA		16 (41)
No GA		23 (59)
*rs429358 (APOE)*		
ε4 ausente		30 (76,9)
ε4 presente		9 (23,1)

DE: desviación estándar; SNP: Polimorfismo de nucleótido simple; ANKK1: *Ankyrin repeat and kinase domain containing 1*; TNF: factor de necrosis tumoral; IL-6: Interleuquina-6; TOP2a: topoisomerasa 2a; BDNF: factor neurotrófico derivado del cerebro; APOE: apolipoproteina E; T presente: alelo T en genotipo; GG: genotipo GG; AG: genotipo AG; GT: genotipo GT; CT: genotipo CT; CC: genotipo CC; GA: genotipo GA; No GA: variante genotipo diferente a GA; ε4 ausente: variante genotipo sin alelo ε4; ε4 presente: variante de genotipo con al menos un alelo ε4.

### Evolución neurocognitiva

La Tabla 2 muestra la mediana de puntuación de la población global para cada prueba neurocognitiva en cada uno de los tres momentos evaluados. En la cohorte global, se identificó empeoramiento estadísticamente significativo de M0 a M1 en dos test: RAVLT (p=0,003) y Letras y números (p=0,013).

**Tabla 2:** Mediana de puntuación en cada test en los tres momentos de evaluación

	Global			Subgrupo SNP		
	n= 62			n= 39		
Test	M0	M1	M2	M0	M1	M2
FAST	31,5	34	39	32	36	44
RAVLT	45	39	45	45	42	50
Clave de números	10	10	12	10	10	13
Cubos	11	11	12	11	11	14
Dígitos	10	10	12	10	11	13
Memoria visual (percentil)	60	60	70	60	70	95
Letras y números	11	9	11	11	10	12,5
Stroop-Interferencia	0,68	3,18	5,35	0,68	3,18	5,35
TMT A (segundos)	40	40	35	40	42	45
TMT B (segundos)	97	90	80	106	100	94

SNP: polimorfismo de nucleótido simple; FAST: test de fluencia verbal semántica; RAVLT: test de Aprendizaje Auditivo-Verbal de Rey; TMT: *Trail Making Test*; M0: basal previo a quimioterapia; M1: hasta cuatro semanas tras finalizar quimioterapia; M2: entre 24-30 semanas tras finalizar quimioterapia.

Tras completar la quimioterapia, las diferencias entre puntuaciones en M2 y en momentos previos (M0 y M1) muestran una tendencia a la mejoría, siendo estadísticamente significativa con respecto a M1 y también a M0 para los test de memoria visual, clave de números, y cubos (Tabla 3).

**Tabla 3:** Diferencia de puntuaciones entre momentos temporales

Test	t-Student		Test	Wilcoxon
	Media de diferencias (DE)	p		p
*FAST*			*Clave de números*	
M0 - M1	0,34 (6,72)	0,705	M1 - M0	0,085
M0 - M2	-4 (8,54)	0,003	M2 - M0	0,001
M1 - M2	-3,58 (6,65)	0,001	M2 - M1	<0,001
*RAVLT*			*Cubos*	
M0 - M1	4,71 (11,26)	0,003	M1 - M0	0,522
M0 - M2	3,36 (12,52)	0,079	M2 - M0	0,001
M1 - M2	-1,31 (9,23)	0,346	M2 - M1	<0,001
*Dígitos*			*Visual*	
M0 - M1	0,42 (2,19)	0,163	M1 - M0	0,33
M0 - M2	-0,69 (2,98)	0,128	M2 - M0	0,012
M1 - M2	-0,78 (3,25)	0,115	M2 - M1	0,009
*Letras y números*			*TMT-A*	
M0 - M1	0,91 (2,61)	0,013	M1 - M0	0,361
M0 - M2	0,07 (2,93)	0,88	M2 - M0	0,236
M1 - M2	-0,8 (3,14)	0,095	M2 - M1	0,617
*Stroop Interferencia*			*TMT-B*	
M0 - M1	0,75 (12,94)	0,667	M1 - M0	0,462
M0 - M2	0,61 (11,39)	0,72	M2 - M0	0,299
M1 - M2	-0,02 (11,31)	0,99	M2 - M1	0,488

DE: desviación estándar; FAST: test de fluencia verbal semántica; RAVLT: test de Aprendizaje Auditivo-Verbal de Rey; TMT: *Trail Making Test*; M0: basal previo a quimioterapia; M1: hasta cuatro semanas tras finalizar quimioterapia; M2: entre 24-30 semanas tras finalizar quimioterapia.

### Análisis de factores clínicos

Los pacientes menores de 55 años obtuvieron mejores puntuaciones medias respecto a aquellos de 55 años o más, fundamentalmente en las pruebas que evalúan la memoria (en los tres momentos en RAVLT y en M2 en el test de memoria visual) y la función ejecutiva (en momentos M1 y M2 de FAST y en todas las evaluaciones de TMT-A y TMT-B).

Los hombres obtuvieron puntuaciones significativamente mejores en los test de cubos y memoria visual en momento basal M0, y en letras y números y Stroop en momento postquimioterapia (M1) (Tabla 4).

**Tabla 4:** Comparación de puntuaciones medias (desviación típica) obtenidas en los distintos test según categorías de variables

Test	Edad		Sexo		Estudios		Comorbilidades	
	<55	≥ 55	Mujer	Hombre	Básicos	Superiores	Sí	No
FAST^a^								
M0	35,9 (10,7)	29,4 (13,8)	18,9(6)	18,7(5,3)	28(11,9)	36,6(12,4)	29,3(11,8)	36,5(13,2)
M1	38,9 (13,2)	28,1 (11,3)	19(7,9)	16,8(4,1)	27,8(10,6)	37,5(13,8)	29,3(11,4)	38,7(14)
M2	43,3 (14,7)	34,1 (10,5)	20,6(6,9)	21,4(4,9)	34,4(9,7)	41,8(15)	34,2(10,3)	44,7(15)
Cubos^a^								
M0	10,8 (2,8)	11,3 (2,4)	10,8 (2,7)	12,4(1,2)	11(2,6)	11,1(2,6)	10,8(2,5)	11,4(2,7)
M1	11,1 (3,7)	10,7 (2,4)	10,7(3,3)	11,9(0,8)	10,5(2,4)	11,2(3,5)	10,2(2,8)	11,8(3,2)
M2	12,7(3,1)	12,4 (2,2)	12,4 (2,8)	13,3(1,6)	12,3(2,4)	12,7(2,9)	12,2(2,2)	13(3,2)
RAVLT								
M0	51,8 (13)	39,4 (12,5)	46,4(15)	38,5(5,7)	42,4(13,7)	47,5(14,3)	42,2(14,3)	48,8(13,1)
M1	46,9 (11,2)	35,4 (10,8)	42(12,9)	34,6(6,3)	38,1(12,6)	42,9(11,9)	36,2(9,9)	47,2(12,7)
M2	48,7 (11,3)	37 (13,6)	43,7(14,4)	39,4(8,9)	42(14,3)	43,7(13,5)	38,6(13,9)	48,5(11,5)
Visual^b^								
M0	56,8 (30)	60,6 (27,4)	55,3(28,9)	75,5(19,4)	55,6(27,3)	62,1(29,5)	61,3(29,2)	55,6(27,5)
M1	59,8 (28,4)	51,4 (32,5)	53,4(30,7)	65,6(29,9)	49,6(32)	59,8(29,3)	53,1(31,2)	58,5(30,3)
M2	77 (23,9)	62,7 (23,9)	68,9(25,7)	75,7(18,8)	64,4(25,4)	73,7(24)	68(26,1)	72,5(23,4)
Letras y números^a^								
M0	9,6 (2,6)	11,4 (2,7)	10,3(2,5)	12,1(3,3)	10,5(3)	10,7(2,5)	8,4(2,9)	8,8(2,8)
M1	9,2 (3,1)	10,2 (2,9)	9,3(3)	12(2,3)	10(3,2)	9,6(2,9)	8,4(3,3)	8,5(3)
M2	10,2 (3,1)	11 (2,9)	10,1(3)	13,1(1,9)	9,8(2,8)	11,1(3,1)	8,3(3,5)	10(2,5)
STROOP^c^								
M0	1,6 (13,16)	2,02(10,94)	0,6(11,48)	7,53(12,67)	-0,35(9,88)	4,02(13,42)	2,1(9,89)	1,46(14,43)
M1	2,11(10,03)	0,61(11,62)	0,22(11,14)	6,96(7,02)	1,53(12,74)	1,16(9,29)	2,08(8,64)	0,27(13,43)
M2	4 (10,73)	-0,21(7,87)	1,58(10,07)	3,94(6,46)	-0.47(9.34)	3,55(9,56)	0,88(8,8)	3,27(10,54)
TMT-A^d^								
M0	35,7 (14,1)	58,8 (25,6)	48,3(23,5)	47,5(27,3)	58,2(27,4)	37,8(13,8)	53(23,6)	41,7(23,3)
M1	36,6 (18)	51,5 (17,9)	44,7(19,8)	43(17,9)	52,7(20,1)	38,1(16,3)	48,3(20,1)	39,2(17,3)
M2	32,5 (13,4)	52,8 (23,7)	44,2(22,9)	32,9(6,7)	51,1(25,7)	36,6(16,2)	48,8(22,5)	34,5(17,7)
TMT-B^d^								
M0	94,3 (61,3)	142,4(102,4)	122,7(92)	109,4(74,4)	152,8(109,8)	86,7(38,7)	130,4(96,6)	106,7(76,5)
M1	76,9 (39,9)	127,2 (70,9)	102,7(61,6)	107,2(74,5)	129,8(77,4)	82,9(39,9)	116,2(65,5)	85,7(56,3)
M2	77,3 (36,8)	118,5 (62,5)	99(58,4)	88,9(26,2)	121,6(65,6)	81,4(39,4)	114,3(57,5)	76,3(43,2)
**Test**	**Tabaco**		**Alcohol**		**Quimioterapia**		**Radioterapia adyuvante**	
	**Sí**	**No**	**Sí**	**No**	**Neo-Adyuvante**	**Adyuvante**	**Sí**	**No**
FAST^a^								
M0	31,7(13,3)	34,7(11)	31(12,2)	32,6(13,1)	36,5(9,8)	30,3(13,7)	35,5(12,5)	29,2(12,6)
M1	32,9(14,1)	34,4(10,4)	31,5(11,1)	33,7(13,9)	39,8(13,5)	29,8(11,9)	37(14,1)	29,4(11,4)
M2	39,9(14,7)	35,5(8,5)	36,2(11,2)	39,6(14,1)	43,9(13,3)	35,8(12,9)	40(14,5)	37,3(12,2)
Cubos^a^								
M0	10,8 (2,7)	12,1(2,1)	11,7(3,4)	10,9(2,4)	11,5(2,9)	10,8(2,5)	11,5(2,9)	10,7(2,2)
M1	10,4(3,2)	12,5(2)	10,7(4)	11(2,8)	11,5(4)	10,6(2,4)	11,4(3,2)	10,4(2,8)
M2	12,3(2,9)	13,5(1,5)	13,1(3)	12,4(2,6)	12,9(3,3)	12,4(2,3)	12,7(3)	12,3(2,1)
RAVLT								
M0	42,9(13,9)	52,8(12,5)	41,8(11)	45,7(14,7)	43,5(15,8)	45,6(13,3)	45,3(16,3)	44,6(11,7)
M1	40,8(12)	41(13,9)	42,8(13,9)	40,3(12)	45,2(12,2)	38,5(11,9)	41,3(13,7)	10,4(10,9)
M2	41,9(14)	46,5(2,7)	45(13,9)	42,4(13,8)	47,1(13,5)	40,5(13,4)	42,8(15,8)	43,2(10,4)
STROOP^c^								
M0	0,21(10,45)	7,93(15,2)	2,39(16,76)	1,7(10,63)	1,72(14,14)	1,88(10,86)	1,73(12,59)	1,93(11,37)
M1	1,74(10,09)	-0,18(13,52)	4,56(11,43)	0,42(10,61)	1,02(8,32)	1,48(12,04)	-0,17(12,18)	2,87(9,18)
M2	0(8,86)	7,96(9,56)	6,14(8,49)	0,74(9,64)	2,43(10,83)	1,65(8,92)	2,56(9,64)	1,09(9,68)
TMT-B^d^								
M0	128,9(96,1)	88,5(41,9)	92,6(31,3)	126,4(96)	93(46,7)	133,6(101)	99,9(63,6)	140(104,9)
M1	112,4(67,4)	71,2(27,6)	96,8(57,5)	105,3(65,2)	91,4(52,2)	109,7(68)	90,7(50,8)	116,6(72,5)
M2	98,2(54,3)	95,1(57,8)	101,4(71,8)	96,3(49,8)	81,7(43,6)	107(58,9)	99(61)	95,4(45,8)

Solo se muestran aquellos test en los que se identifica al menos una diferencia significativa (resaltada en negrita); a: puntuación estimada de acuerdo a edad y nivel educativo; b: percentil ajustado a edad y nivel educativo; c: interferencia; d: puntuación directa (segundos); FAST: test de fluencia verbal semántica; RAVLT: test de Aprendizaje Auditivo-Verbal de Rey; TMT: *Trail Making Test*; M0: basal previo a quimioterapia; M1: hasta cuatro semanas tras finalizar quimioterapia; M2: entre 24-30 semanas tras finalizar quimioterapia.

La función ejecutiva y la atención son los dominios más relacionados con el nivel de estudios; las personas con estudios superiores obtuvieron mejores resultados en TMT-B (todas las evaluaciones) y en FAST (M0 y M1).

La ausencia de comorbilidades se asocia con mejores puntuaciones en todas las evaluaciones de FAST y RAVLT, así como en función ejecutiva y atención medidas mediante TMT tras quimioterapia (M1) (Tabla 4).

En cuanto al consumo de tóxicos, la puntuación media fue significativamente superior en pacientes no fumadores en RAVLT basal y Cubos en M1, lo que implica menor impacto de la quimioterapia sobre las aptitudes visuales*.* Aquellos pacientes sin consumo de tóxicos presentaron mejores resultados en la última evaluación (M2) de Stroop, tanto para alcohol (p=0,038) como para tabaco (p=0,024) (Tabla 4).

Los pacientes que reciben quimioterapia neoadyuvante presentaron mejores puntuaciones en FAST en momento post-quimioterapia (p=0,01) con respecto a aquellos que reciben quimioterapia adyuvante. La radioterapia adyuvante se asoció con mejores resultados en FAST tanto en momento M0 (p=0,046) como en M1 (p=0,048) (Tabla 4).

### Análisis de factores genéticos

Los seis polimorfismos estudiados se asociaron con diferencias significativamente distintas en las puntuaciones de alguno de los test (Tabla 5).

**Tabla 5:** Comparación de puntuaciones medias (desviación típica) según categorías de variantes de polimorfismo de nucleótido simple (SNP)

Test	rs1800497		rs1800629		rs1800795		rs471692	
	T -	T +	GG	AG	GT	GG	CC	CT
*FAST*^*a*^								
M0	35,4(12)	34,9(15,1)	36,8(13)	32,3(13,2)	30,9(14,3)	38,5(11,3)	37,5(13,2)	27,3(9,5)
M1	35,7(15,1)	33,4(12)	36,4(14,2)	32(13,2)	32,7(14,9)	36,4(13,1)	37(14,4)	27,4(9)
M2	40,5(15)	41,4(8,5)	342,6(14,1)	38,1(11,2)	41(14,4)	40,6(12,4)	42,9(13,2)	34,4(10,5)
*RAVLT*								
M0	50(15,2)	42,5(9,6)	51,1(11,9)	39,9(14,1)	45,6(16,2)	48,3(11,6)	48,1(13,8)	43,9(13,7)
M1	46,4(13,2)	36,9(9,9)	44,4(12,8)	39,9(12,7)	43,4(12,9)	42,3(13)	44,4(13,1)	37,3(10,7)
M2	45,3(15,9)	45,8(7,5)	47,7(11,1)	42,2(16,3)	42,8(14,1)	47,3(13,1)	48,2(13)	37,3(12)
*Visual*^*b*^								
M0	54(29,2)	74(17)	63,8(25,6)	57,9(29,5)	62,4(24,2)	61,1(29,3)	65(27,3)	50,6(23,5)
M1	59,4(28,9)	62,7(26,2)	64,2(25,5)	54,3(31,1)	61,2(30,7)	60,2(25,7)	64(24,6)	49,4(35,3)
M2	69,8(26,5)	75,5(20,4)	78,7(24,1)	61,5(21,8)	76,9(20,7)	68,2(26,6)	77,1(19,8)	55,6(30,8)
*STROOP*^*c*^								
M0	1,74(9,8)	2,32(14,33)	-0,44(10,44)	6,25(12,61)	0,83(11,35)	2,83(11,93)	0,79(12,57)	5,87(6,39)
M1	1,1(8,34)	0(15,19)	-2,49(12,01)	6,35(7,23)	0,08(10,24)	1,14(12,25)	0,12(12,5)	2,53(5,85)
M2	-0,64(8,52)	5,04(7,39)	2,29(8,3)	-0,11(8,87)	0,81(9,81)	1,66(7,71)	2,01(7,33)	-0,78(11,63)
*TMT-A*^*d*^								
M0	42,4(18,8)	46,9(24,8)	39,8(16)	51,7(27,1)	44,9(23)	43,5(20,1)	39,6(16,5)	59(28,4)
M1	41,9(19,5)	41,3(16,4)	38,6(14,1)	47,1(23,4)	40,1(19,1)	42,8(17,8)	37,5(15,4)	55,6(20,6)
M2	42,1(21,8)	38,1(21,1)	36,7(18,3)	46,5(24,6)	32,9(13,9)	46,1(24)	36,6(19,6)	53,3(22,5)
*TMT-B*^*d*^								
M0	109,9(66,6)	101,1(60,1)	90,3(36,1)	135,5(89,3)	124,9(69,7)	92,3(55,8)	87,2(42,8)	170,8(80,5)
M1	88,9(44,5)	97,2(63,4)	78,6(31,7)	116,3(71)	99,1(62,4)	86,7(42,9)	80,5(35,9)	130,8(77,1)
M2	90,1(49,7)	95,6(54,4)	81,1(45,8)	108,1(54,6)	78,8(34,7)	101,1(58,2)	81,7(44,9)	123(56,8)
*Clave de números*^*a*^								
M0	10,5(2,4)	10,3(2,3)	10,8(2,5)	9,9(2)	10,2(2,4)	10,6(2,3)	10,6(2,5)	9,8(1,4)
M1	10,4(3,5)	9,9(1,6)	10,6(2,9)	9,6(2,8)	10,2(3,1)	10,2(2,8)	10,4(2,6)	9,4(3,6)
M2	11,9(2,8)	12,7(2,7)	12,7(3)	11,5(2,2)	12,2(3,5)	12,2(2,2)	12,8(2,8)	10,5(1,5)
	**rs6265**		**rs429358**					
**Test**	**No GA**	**GA**	**ε4 ausente**	**ε4 presente**				
*STROOP*^*c*^								
M0	1,4(8,71)	2,76(15,06)	1,21(9,82)	4,45(16,64)				
M1	-0,98(12,02)	3,07(10,03)	-1,18(9,91)	6,89(13,88)				
M2	0,39(7,8)	2,85(9,66)	-0,51(7,63)	6,8(9,01)				
*TMT-A*^*d*^								
M0	39,4(16,8)	50,9(25,2)	45,1(22,5)	40,8(16,4)				
M1	35,8(12,4)	50(22)	42,7(17,9)	38,1(19,6)				
M2	36,2(17,6)	48,3(25,3)	42,5(21,1)	35,5(22,3)				

Solo se muestran aquellos test en los que se identifica al menos una diferencia significativa (resaltada en negrita); a: puntuación estimada de acuerdo a edad y nivel educativo; b: percentil ajustado a edad y nivel educativo; c: interferencia; d: puntuación directa (segundos); FAST: test de fluencia verbal semántica; RAVLT: test de Aprendizaje Auditivo-Verbal de Rey; TMT: *Trail Making Test*; M0: basal previo a quimioterapia; M1: hasta cuatro semanas tras finalizar quimioterapia; M2: entre 24-30 semanas tras finalizar quimioterapia.

En el momento basal, algunas variantes se asociaron con resultados significativamente mejores: la variante CC de rs471692 en el test FAST, la variante GG de rs11800629 en el test RAVLT, y la T+ de rs1800497 en el test de memoria visual (Tabla 5).

Los polimorfismos genéticos rs1800629 (TNFα) y rs471692 (TOP2A) fueron los que más se relacionaron con el rendimiento cognitivo.

El genotipo CC del rs471692 en TOP2A se relacionó con mejor rendimiento en función ejecutiva y atención en todos los momentos evaluados, y el genotipo GG de rs1800629 con puntuaciones significativamente mejores en memoria y función ejecutiva que aquellos con variante AG.

Los SNP rs1800497, rs1800795, rs6265 y rs429358 únicamente se asociaron con diferencias estadísticamente significativas en las puntuaciones de un test (Tabla 5).

### Análisis multivariante

En momento M0, los factores significativamente asociados a mejores resultados en función ejecutiva (evaluada por FAST) fueron nivel de estudios superior, rs471692 y rs1800795; a mejores resultados de memoria (RAVLT) fueron rs1800629 y presencia de alelo T en rs1800497. En el test de Stroop únicamente fue el sexo masculino y en atención (medida mediante TMT-A) la relación significativa se identificó en aquellos con edad menor de 55, sin comorbilidades, con sexo femenino, rs1800497 y rs6265.

En M1, los factores que mejor predijeron el rendimiento cognitivo en FAST fueron el nivel de estudios superior y la quimioterapia neoadyuvante; en RAVLT la presencia de alelo T de rs1800497. Los sujetos menores de 55 años, de sexo masculino, sin comorbilidades y que no reciben radioterapia adyuvante además de los que presentan polimorfismos rs1800629 y rs1800497 obtuvieron mejores resultados en función ejecutiva, evaluada con Stroop. En TMT-A, la asociación con mejores resultados se identificó entre los pacientes jóvenes y sin comorbilidades que no consumen alcohol y con rs6265 (Tabla 6).

**Tabla 6:** Análisis multivariante de regresión lineal de los factores de riesgo asociados con empeoramiento cognitivo

Test	Tiempo	Variable	B*	IC95%	p
FAST	M0	Nivel estudios (básico)	-8,338	-15,889 a -0,788	0,031
		rs1800795 (GT)	-7,517	-15,002 a -0,033	0,049
		rs471692 (CC)	-9,746	-18,561 a -0,931	0,031
	M1	Nivel estudios (básico)	-9,42	-17,418 a -1,422	0,022
		QT (adyuvante)	-8,748	-0,338 a -17,158	0,042
		rs471692 (CT)	-7,873	-16,971 a 1,224	0,088
	M0-M1	Edad (≥ 55)	-0,283	-0,513 a -0,053	0,018
		Sexo(masculino)	-10,386	-17,752 a -3,019	0,007
		Alcohol (consumo)	-5,28	-10,842 a 0,282	0,062
		QT (adyuvante)	-6,358	-10,949 a -1,767	0,008
		rs1800497(T+)	-4,922	-10,001 a -0,157	0,057
		rs1800795 (GT)	-5,584	-9,906 a -1,262	0,013
RAVLT	M0	Tabaco (consumo)	-7,879	-16,792 a 1,033	0,081
		rs1800497(T+)	-10,13	-18,052 a -2,175	0,014
		rs1800629(AG)	-11,356	-19,484 a -3,228	0,008
	M1	Edad (≥ 55)	-0,743	-1,112 a -0,373	0,001
		Sexo(masculino)	-10,406	-21,65 a 0,837	0,069
		Nivel estudios (básico)	-6,173	-13,541 a 1,196	0,098
		QT (adyuvante)	-6,286	-13,606 a 1,034	0,090
		rs1800497(T+)	-12,399	-20,550 a -4,249	0,004
	M0-M1	Tabaco (consumo)	-9,645	-17,441 a -1,849	0,017
		Alcohol (consumo)	-9,190	-17,108 a -1,272	0,024
		QT (adyuvante)	-7,017	-14,085 a 0,051	0,052
		rs1800629 (AG)	-5,98	-13,063 a 1,104	0,095
STROOP	M0	Sexo (masculino)	-16,358	-27,983 a -4,733	0,007
		rs1800497(T-)	-6,36	-15,317 a 2,597	0,158
		rs1800795 (GT)	-4,35	-11,693 a 2,993	0,237
	M1	Edad (≥ 55)	-0,649	-0,970 a -0,328	0,000
		Sexo(femenino)	-14,138	-24,951 a -3,324	0,012
		Comorbilidades(no)	-8,219	-14,238 a -2,200	0,009
		RT (ausencia)	-6,073	-12,595 a 0,449	0,067
		rs1800497(T+)	-8,176	-15,776 a -0,576	0,036
		rs1800629 (GG)	-9,347	15,949 a -2,745	0,007
	M0-M1	Edad (≥ 55)	-0,213	-0,616 a 0,191	0,291
		QT (adyuvante)	-5,016	-15,761 a 5,729	0,350
		RT (no)	-9,389	-19,359 a 0,580	0,064
TMT-A**	M0	Edad (≥ 55)	1,702	1,213 a 2,190	0,000
		Sexo(femenino)	20,551	4,574 a 36,527	0,013
		Comorbilidades(presencia)	15,616	5,923 a 25,309	0,002
		rs1800497(T+)	13,051	1,352 a 24,751	0,030
		rs6265 (GA)	7,966	-1,295 a 17,227	0,089
	M1	Edad (≥ 55)	0,989	0,572 a 1,405	0,001
		Comorbilidades(presencia)	7,999	-0,494 a 16,492	0,064
		Alcohol (no consumo)	10,848	0,785 a 20,911	0,035
		rs6265 (GA)	12,728	4,379 a 21,078	0,004
	M0-M1	Comorbilidades(sí)	8,023	-0,053 a 16,100	0,051
		QT (adyuvante)	7,508	-0,919 a 15,935	0,079

En negrita los resultados significativos; FAST: test de fluencia verbal semántica; RAVLT: test de Aprendizaje Auditivo-Verbal de Rey; TMT: *Trail Making Test*; M0: basal previo a quimioterapia; M1: hasta cuatro semanas tras finalizar quimioterapia; M2: entre 24-30 semanas tras finalizar quimioterapia; *: variación en la puntuación del test neurocognitivo; **: mayores puntuaciones implican mayor deterioro.

Se encontró asociación estadísticamente significativa entre los cambios cognitivos observados tras el tratamiento de quimioterapia en FAST en relación a la edad, sexo, tipo de quimioterapia y rs1800795. También en RAVLT según consumo de tabaco y alcohol. No se encontraron diferencias estadísticamente significativas en este análisis multivariante en el resto de pruebas valoradas (Tabla 6).

## DISCUSIÓN

Los resultados del presente trabajo aportan nuevas evidencias para la identificación de factores de riesgo de CRCI (clínicos, genéticos y/o asociados a la enfermedad oncológica), así como de los dominios neurocognitivos más afectados en un grupo de pacientes con tumores de mama y colon que reciben tratamiento con quimioterapia.

En 2004, Wefel y col realizaron el primer estudio longitudinal que mostró que hasta el 33% de las pacientes con cáncer de mama presentaban, antes de recibir tratamiento sistémico, déficits cognitivos^6^. Con esta base y, ante la posibilidad de que el declive cognitivo se inicie incluso antes de recibir el tratamiento, la evaluación basal (previa al inicio de quimioterapia) resulta fundamental. Realizamos también una evaluación inmediatamente posterior al fin de la quimioterapia y una evaluación a largo plazo lo que permite valorar el impacto de los diferentes factores estudiados en cada uno de los momentos.

La edad es un factor de riesgo demostrado en el desarrollo de deterioro cognitivo, siendo la velocidad de procesamiento y la memoria los dominios que parecen más afectados^24^. En nuestro estudio se confirma una relación importante entre la edad y el CRCI. Además, tras el análisis multivariante y, de manera similar a lo reportado en revisiones previas^25^, la edad impacta fundamentalmente en atención y función psicomotora medida mediante el test TMT-A.

Los cambios hormonales producidos por la quimioterapia afectan fundamentalmente a las hormonas sexuales femeninas por lo que se plantea la posibilidad de que el sexo pueda influir en el desarrollo de CRCI^26^. En trabajos previos, el sexo femenino parece impactar negativamente sobre todo en la evaluación basal, y la memoria verbal es el dominio más afectado^27^, mientras que en nuestro trabajo la mayor asociación es con la función ejecutiva.

La reserva cognitiva basal se ha relacionado con el deterioro post-tratamiento, presentando los pacientes con mayor nivel de estudios mejores resultados en los diferentes momentos temporales^28^. En nuestro trabajo dicha relación se detecta fundamentalmente en evaluación basal.

El deterioro cognitivo se ha asociado con la alta prevalencia de comorbilidades en pacientes con cáncer de mama^29^, y con el consumo de tabaco y de alcohol de forma generalizada^30^. Aunque en nuestro estudio la relación entre alcohol y CRCI se ha registrado independientemente de la intensidad del consumo, está descrito que el impacto del alcohol parece depender de la cantidad diaria consumida^31^. Nuestros datos confirman la tendencia de una asociación entre comorbilidades y tabaco y peores resultados en test de atención. No obstante, en este estudio tan solo el 21% de los pacientes eran fumadores en el momento de la inclusión, lo que representa un porcentaje inferior al de otras publicaciones^29^.

La predisposición genética es uno de los factores de riesgo de deterioro cognitivo asociado a cáncer menos estudiados.

APOE está implicada en la plasticidad neuronal y en los mecanismos de reparación tras agresiones. La presencia de al menos un alelo Ɛ4 de APOE se ha asociado con mayor vulnerabilidad al deterioro cognitivo inducido por quimioterapia^32^. En nuestro estudio, los portadores de alelos Ɛ4 de APOE presentaron peores resultados que los no portadores en test de atención tras el tratamiento con quimioterapia. La misma tendencia se detectó en los portadores de la variante CT en rs471692 lo que podría indicar un mayor impacto de la quimioterapia sobre la atención en estos subgrupos de pacientes. No obstante, dado el pequeño tamaño muestral, estos resultados deben interpretarse con cautela y confirmarse en una muestra mayor.

La evidencia sobre la relación entre variantes de BDNF, concretamente Val66Met, rs1800795 y CRCI es más limitada^33^^,^^34^. Nuestro análisis está en línea con otros trabajos que asocian el alelo G del SNP de IL-6 con peores resultados en test de atención. A pesar de que IL-6 tiene un papel muy importante en el deterioro cognitivo, los polimorfismos de IL-6 no se relacionan de manera significativa con el deterioro cognitivo^35^.

En el análisis multivariante, y a pesar del pequeño tamaño muestral, los SNPs se confirman como potenciales factores implicados en los cambios neurocognitivos.

Nuestro trabajo cuenta con fortalezas y limitaciones. El diseño prospectivo y longitudinal permite valorar las diferencias en capacidades neurocognitivas en función de diferentes factores individuales. Aporta información obtenida en práctica clínica para optimizar la identificación de aquellos sujetos con mayor riesgo de CRCI, lo que permitiría adaptar las decisiones terapéuticas y realizar determinadas intervenciones encaminadas a atenuar el impacto del deterioro en estos pacientes. Además, el empleo de una amplia batería de pruebas objetivas validadas de acuerdo a las recomendaciones de ICCTF^5^, junto con el diseño prospectivo longitudinal, permite realizar un seguimiento y una evaluación homogénea de todos los sujetos incluidos. Entre las limitaciones se encuentran que se trata de una serie pequeña con una tasa de abandono superior a lo esperado (27,5%), y la ausencia de grupo control que limita la posibilidad de conocer específicamente el rol del cáncer y sus tratamientos en los resultados neurocognitivos identificados. Por todo esto, los resultados deben interpretarse con precaución.

Sin embargo, este estudio destaca por explorar de manera longitudinal la relación entre diversos factores genéticos y clínicos, así como su impacto sobre el estatus neurocognitivo en una cohorte de pacientes tratados con quimioterapia, lo que ha permitido identificar diversos factores de predisposición a CRCI. Una mayor edad, el sexo femenino, la presencia de comorbilidades y el nivel básico de estudios se relacionan con un mayor riesgo de CRCI, afectando especialmente a la memoria y a la función ejecutiva. Los genotipos CC de rs471692, GG de rs11800629 y T+ de rs1800497 se asociaron a un mejor estatus neurocognitivo en el momento basal, mientras que aquellos con variante CT de rs471692 y los portadores de un alelo ε4 de rs6265 presentaron mayor deterioro en atención.

Estos conocimientos nos permiten identificar el impacto de diferentes factores sobre las aptitudes neurocognitivas y la aplicabilidad de SNPs como biomarcadores para la creación de subgrupos considerados de riesgo para el desarrollo de CRCI.
